# Titanium migration and bone response in loaded osseointegrated implants: ESEM‐EDX analysis in *Macaca fascicularis*

**DOI:** 10.1002/jper.70003

**Published:** 2025-09-11

**Authors:** Fausto Zamparini, Andrea Spinelli, Maria Giovanna Gandolfi, Stefano Chersoni, Achille Tarsitano, Giovanni Badiali, Chooi Gait Toh, Carlo Prati, Georgios Romanos

**Affiliations:** ^1^ Endodontic Clinical Section Dental School DIBINEM University of Bologna Bologna Italy; ^2^ Laboratory of Biomaterials and Oral Pathology Dental School DIBINEM University of Bologna Bologna Italy; ^3^ Oral and Maxillo‐Facial Surgery Unit DIBINEM University of Bologna Bologna Italy; ^4^ School of Health Sciences International Medical University Kuala Lumpur Malaysia; ^5^ Department of Periodontics and Endodontics School of Dental Medicine Stony Brook University Stony Brook New York USA; ^6^ Department of Oral Surgery and Implant Dentistry Dental School (Carolinum) Johann Wolfgang Goethe University Frankfurt Germany

**Keywords:** bone remodeling, dental implants, energy‐dispersive X‐ray spectroscopy, environmental scanning electron microscopy, peri‐implant bone, titanium migration

## Abstract

**Background:**

Titanium nanoparticle (TP) migration into peri‐implant bone may influence osseointegration. It remains unclear how loading protocols may affect TP distribution. This study aimed to detect TP in the bone around implants undergoing different loading protocols in *Macaca fascicularis*.

**Methods:**

Nine histological samples containing 21 implants with two loading groups were analyzed. In the delayed‐loaded (DL) group (*n* = 16), the implants were loaded after 3 months and retrieved after 3 months, and in the immediately loaded (IL) group (*n* = 5), they were loaded on the day of surgery and retrieved after 3 months. Environmental scanning electron microscopy (ESEM) grayscale‐level detection and energy‐dispersive X‐ray spectroscopy (EDX) microchemical analysis were used to assess TP and bone mineralization. Regions of interest (ROI) located at the implant coronal/apical portion (100×) and at the bone–implant interface (1000×) were selected. Bone area distribution (mean% ± SD%) and titanium content were analyzed using two‐way analysis of variance (ANOVA) (*p* < 0.05).

**Results:**

Titanium granules (2–10 µm) were detected in all regions, with a higher prevalence in the coronal portions of DL implants. In IL implant sections, bone closer to the implants showed a lower prevalence of titanium (*p* < 0.05). EDX analysis demonstrated a decreasing trend in titanium from the nearest areas to those more distant (up to 2.0 mm). DL implants exhibited lower percentages of mineralized bone compared to IL implants in the coronal portion (mean values 31.0 ± 13.7 and 11.6 ± 2.8) (*p* < 0.05). IL implants showed a higher percentage of mineralized bone (*p* < 0.05) in the apical region (mean values 51.8 ± 15.5 and 32.2 ± 15.6).

**Conclusion:**

TP were widely present in bone tissues adjacent to the implant surface, particularly at the coronal bone. In the coronal portion of the DL group, a less mineralized bone area was observed compared to the IL group, suggesting higher bone remodeling activities.

**Plain Language Summary:**

Titanium particles were widely present in bone tissues adjacent to the implant areas, with greater distribution observed in regions experiencing significant wear (i.e., the coronal portion of the cortical bone), likely due to surgical insertion and related procedures.

## 1 INTRODUCTION

Stable peri‐implant bone levels are a necessary condition for long‐term health of dental implants.^1^, ^2^, ^3^, ^4^, ^5^ However, physiological bone remodeling is expected to occur in the first months after insertion. During this period, implants are at high risk of failures due to biomechanical instability or insufficient osseointegration.^1^, ^6^

Loading procedures are a further critical step that could influence crestal marginal bone level and long‐term survival of the implant^5^ as a higher remodeling activity is present around the implant.^7^, ^8^, ^9^, ^10^ Different loading timings are reported in literature.^11^ Conventional procedures include a 3–6‐month healing period before the placement of a prosthetic crown, while immediate protocols allow the placement of an immediate prosthesis at the moment of implant surgery. A recent meta‐analysis revealed that immediate loading, compared with conventional loading, could be associated with a higher incidence of early implant failures.^12^ As a consequence, these two protocols may provide a very different scenario with different bone remodeling activities.^5^

The presence of titanium (Ti) particles in the peri‐implant bone and soft tissues has been documented in a number of histological studies.^13^, ^14^, ^15^, ^16^ Possible hypotheses on the mechanisms of Ti nanoparticle migration have been reported in reviews^17^ and laboratory investigations.^18^, ^19^ In the oral cavity, Ti implants are susceptible to corrosion despite the biocompatibility provided by the external oxide layer.^20^ The presence of mechanical wear could breach or remove the layer, resulting in the release of corrosion products and metal ions into the peri‐implant bone or soft tissues (tribocorrosion). Recent studies found high levels of Ti ions in submucosal plaque^21^ or in desquamated cells of the peri‐implant mucosa^22^ of dental implants with peri‐implantitis, possibly indicating a direct correlation between peri‐implantitis and Ti dissolution.^21^, ^23^ However, the detection of Ti particles or ions in the bone tissues has been documented also in healthy sites (not affected by peri‐implantitis).

These events are not a prerogative of dental implants but are also described in other medical surgeries. Previous demonstrations of Ti or other metal element migration into bone tissues have been widely documented in orthopedics and in load‐bearing spinal implants.^24^, ^25^ A recent study investigated the wear and corrosion of Ti alloy spinal implants in vivo. Metal particles were found in both soft and bone tissues close to the surgical sites. The migrated particles revealed good histocompatibility and noninflammatory reactions, demonstrating that a certain degree of corrosion and wear occurs with no bone loss or osteolysis.^24^

Currently, no information is available regarding the effect of initial loading on Ti particles present in peri‐implant bone and the exact location of Ti wear around osseointegrated dental implants.

Environmental scanning electron microscopy (ESEM) has proven effective for examining bone tissue morphology and detection of metal particles. It has been extensively applied to study the bone–implant interface in human specimens.^4^, ^26^, ^27^, ^28^, ^29^, ^30^

Energy‐dispersive X‐ray spectroscopy (EDX) is a rapid, nondestructive, and semiquantitative analytical technique that enables the elemental analysis of delicate or fragile samples while minimizing manipulation, damage, and contamination. EDX has been employed to evaluate bone mineralization around dental implants, providing information on the composition of mineralized tissues.^27^, ^28^, ^29^, ^30^ EDX has proven valuable in detecting the migration of elements, such as iron or Ti, from implanted materials into surrounding bone tissue.^28^

This in vivo study aimed to assess Ti particle migration and bone mineralization activity around osseointegrated implants subjected to immediate‐loading and delayed‐loading protocols in *Macaca fascicularis* using ESEM‐EDX analysis.

## 2 MATERIALS AND METHODS

In this animal study, implants were placed in healed mandibular extraction sites of monkeys using a split‐jaw design, one side serving as control and the contralateral side as the test group. After prosthetic restoration and a 3‐month loading period, the animals were euthanized and bone blocks were harvested for histological analysis. Detailed information on the surgical procedures has been presented in a previous paper.^31^ The study timeline is reported in Table 1.

**TABLE 1:** Study timeline.

Time (weeks)	Event
Week 0	Tooth extractions (bilateral molars and premolars)
Weeks 1–12	Healing period (3 months), oral hygiene 3×/week
Week 12	Control implants (delayed‐loaded group)
Weeks 12–24	Control implant healing (3 months)
Week 24	Test implants (immediately loaded group)
	Delayed‐loaded group: abutments placed (both groups), impressions taken
Week 25	Provisional resin splinted crowns installed (both groups)
Week 29	Final metal splinted crowns installed
Weeks 29–41	3‐month prosthetic loading
Week 41	Sacrifice and bone block harvest for histology

### 2.1 Implant design

The study used an implant system (Ankylos; Dentsply Sirona, Bensheim, Germany) with a progressive thread design made of pure Ti (grade 2), featuring a 2.0‐mm polished collar and a sandblasted surface. Implants were 8.0 mm in length and 3.5 mm in diameter, with straight prefabricated abutments having a 1.0‐mm sulcular collar and a 2.5‐mm height for adaptation to the dentition of *M. fascicularis*.

### 2.2 Animals

Six male *M. fascicularis* (mean weight 5.44 ± 0.61 kg, mean age 7.25 ± 0.5 years), housed individually under controlled conditions at the University of Malaya's Laboratory Animal Research Centre (Kuala Lumpur, Malaysia), were used. They were fed a standard diet of fruits and high‐protein primate biscuits, supplemented with vitamins according to the standard animal care protocol of the Animal House, University of Malaya, Kuala Lumpur, Malaysia. This study was approved by the animal ethics committee of the University of Malaya, Kuala Lumpur, Malaysia (Ethics Ref. No. U1/10/6/96/TCG (R)‐1) and supported financially by a grant from the Ministry of Science, Technology, and Environment, Malaysia. The animals were housed and fed.

### 2.3 Surgical procedure and loading protocol

The monkeys were sedated (tiletamine hydrochloride 4–6 mg/kg) for tooth extraction and implant placement. All mandibular second premolars and first and second molars were sectioned using high‐speed diamond burs and carefully extracted without flap elevation using a thin individually fabricated chisel, followed by clinical and radiographic healing assessments after 3 months. An oral hygiene protocol, including toothbrush and prophylaxis, was carried out three times a week under sedation to maintain healthy periodontal tissues. Three months later, the extraction sites had completely healed, as confirmed by clinical and radiological examination.

**FIGURE 1:**
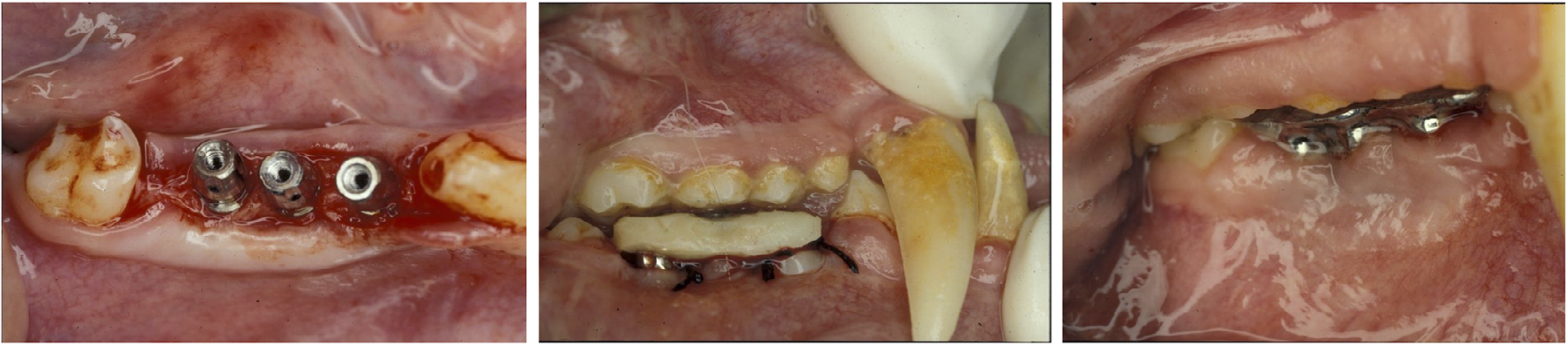
Clinical images of surgical procedures (from left to right): Implant insertion and immediate loading with abutments and provisional resin splinted crowns, followed by definitive rehabilitation with metal splinted crowns.

A split‐jaw design was used with implants on one side of the jaw of each monkey serving as control group and the implants in the contralateral side serving as test group (Figure 1).

Group A (control): Three implants were placed into the healed sockets of one side of the mandible using mucoperiosteal flap elevation and rotary machines operating at 800–1000 rpm with sterile saline irrigation. The torque values were approximately 15 Ncm. After a 3‐month healing period, the implants were surgically exposed, and healing abutments were placed to shape the peri‐implant soft tissue. After 1 week, the abutments were connected and an impression taken together with the test side for fabrication of screw‐retained metal splinted crowns. Provisional screw‐retained resin splinted crowns were placed. After 1 month, they were replaced with screw‐retained metal splinted crowns.

Group B (test): On the day when the abutments were placed in Group A, three implants were placed into the edentulous area on the contralateral side of the mandible. The torque values were approximately 15 Ncm. The abutments were installed and impressions taken at the same time as the Group A abutments for laboratory fabrication of screw‐retained metal linked crowns. Provisional screw‐retained resin splinted crowns were installed for immediate loading of the implants. One month later, the provisional crowns were replaced by metal splinted crowns.

The monkeys were euthanized after 3 months of loading, and the bone blocks were histologically processed. Detailed information on the surgical and prosthetic protocol has been described in Romanos et al. (2002).^32^

### 2.4 Histological biopsy preparation

Detailed information on the histological processing has been provided in previous papers.^31^, ^32^ Briefly, the specimens were immersed in 4% formalin and, after dehydration, embedded in resin according to the Sage–Schiff technique described by Donath and Breuner (1982).^33^ Three slices of approximately 10 µm thickness for each implant were prepared and stained with toluidine blue.

### 2.5 Optical microscopy and ESEM‐EDX microanalysis

The cover slides were removed as follows. Briefly, a cryo‐spray was applied to cool the slide glass. Then, with the aid of a scalpel, the glass slide was lifted and removed in a single step. Subsequently, the samples were immersed in a solution of pure acetone for 1 h, followed by rinsing with pure alcohol and distilled water. Any strong chemical solutions (as xylene) were avoided in order to limit alteration of the fixed specimens.

Optical microscopy (OM) was used to assess the histological sample integrity and to identify the peri‐implant bone morphology. The biopsies were then placed on the ESEM stub and examined without any previous preparation (uncoated samples). Operative parameters were established as follows: low vacuum of 100 Pa, accelerating voltage of 20–25 kV, working distance of 8.5 mm, and a 133‐eV resolution in quadrant back‐scattering detector mode (0.5 wt% detection level, amplification time 100 µs, measuring time 60 s).

### 2.6 Identification of regions of interest

ESEM images at 100× magnification were observed and acquired to assess the overall morphology of peri‐implant bone. Two regions of interest (ROI), each measuring 1.5 × 2.5 mm, were selected in the cortical and apical portions of the implants (see Figure S1 in online *Journal of Periodontology*). These areas were systematically selected in correspondence with the first and the last implant thread with bone tissue.

**FIGURE 2:**
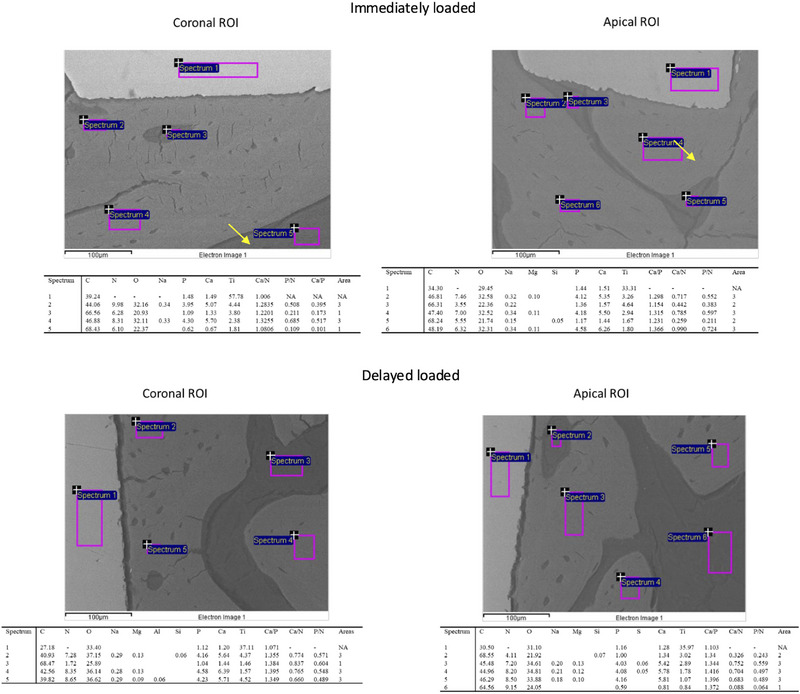
EDX analysis (1000× magnification) of representative immediately loaded and delayed‐loaded implant coronal and apical ROI. ESEM‐EDX analyses enabled calculation of Ca/P, Ca/N, and P/N atomic ratios, which made it possible to distinguish bone areas with different grayscale intensities and mineralization trends. Arrows indicate presence of some light electron‐dense granules belonging to implant thread. Ca, calcium; EDX, energy‐dispersive X‐ray spectroscopy; ESEM, environmental scanning electron microscopy; N, nitrogen; NA, not applicable; P, phosphorus; ROI, region of interest.

To analyze the interfacial bone, the most coronal and apical thread of each available implant was also examined using ESEM‐EDX at 1000× magnification.^27^, ^28^ Microchemical analysis and elemental mapping (calcium [Ca], phosphorus [P], nitrogen [N], and Ti) were performed using EDX. High magnifications are strongly recommended as they allow for more accurate detection of small, electron‐dense granules and for detecting external elements.^27^, ^28^ At least five spectra per ESEM image were acquired. The mineral content was measured with ZAF correction in areas of approximately 30 × 30 µm, and qualitative and semiquantitative element content (weight % and atomic %) was evaluated (Figure 2). For all spectra, the presence of Ca, P, and N and their relative atomic ratios (Ca/P, Ca/N, and P/N) were calculated. EDX mapping was used to detect the elemental distribution of Ca, P, N, and Ti in the selected areas.

### 2.7 Bone area assessment through grayscale intensity quantification

In each ESEM image, areas with different gray intensities (electron density) were identified. ImageJ software (National Institutes of Health [NIH], Bethesda, Maryland, USA) provided grayscale intensity values, ranging from dark to light, to define bone areas with varying elemental concentrations and morphology. Bone 1 corresponded to the lowest electron density (dark gray), while Bone 4 corresponded to the highest electron density (light gray). EDX mapping was further performed to analyze the mineralization gradient. The grayscale intensity was correlated with EDX data following a previously established methodology.^27^, ^28^, ^30^

The extension of different electron‐dense bone areas was measured using ImageJ, calibrated with the ESEM scale bar. Measurements were taken for each ROI, and the mean values (in µm^2^) were recorded. For each ROI, percentages of bone areas were calculated.

### 2.8 Analysis of Ti content in peri‐implant bone

The presence of Ti in bone tissue was evaluated using both ESEM and EDX analyses. ImageJ software was used on the acquired ESEM images to set a threshold for gray intensity. EDX spectra were acquired in the selected ROI (from the implant thread to the most distant bone regions) to quantify the Ti atomic percentages in each spectrum. The distribution of electron‐dense particles was analyzed in each ESEM image. EDX analysis of Ti atomic content was performed in each ROI, namely close to the implant thread (within 300 µm), remote bone (from 300 µm to 2.0 mm), and at distant bone (over 2.0 mm). Apical bone distance of more than 2.0 mm was used as a control group, being unaffected by any surgical procedure.

The elemental detection provided by EDX analysis has been standardized before each ESEM‐EDX session by the software used (Inca software). To avoid artifacts and ensure the accuracy of Ti detection, we performed background and EDX measurements on control regions (out of the histological slides and at distant bone areas, i.e., at a distance of more than 2.0 mm). No Ti was detected in these control measurements. Additionally, we confirmed that the ESEM‐EDX probe and chamber components did not contain Ti or release a Ti signal under the operative parameters used.

### 2.9 Statistical analysis

Two‐way analysis of variance (ANOVA) followed by a Holm–Sidak test (normality test *p* > 0.05, equal variance test *p* > 0.05) was performed to detect statistically significant differences in bone area distribution between loading groups at both coronal and apical ROI. Similarly, Ti atomic content was analyzed using two‐way ANOVA followed by a Holm–Sidak test (normality test *p* > 0.05, equal variance test *p* > 0.05) to detect any statistically significant differences between loading group and ROI. The *p* value was set in all cases at 0.05.

## 3 RESULTS

A total of nine histological slides (bone blocks) were available for the analysis from the previous study. Among these, four slides contained three implants each, four slides contained two implants each, and one slide contained a single implant. In total, 21 implants were evaluated across the nine histological samples. Of these, 15 were delayed‐loaded implants and six were immediately loaded implants. Table 2 reports the bone area percentages at the interface region of coronal and apical ROI. The proportion of bone with low mineralization (Bone Area 1) was higher in the delayed‐loaded group, particularly in the apical region (33.0% ± 13.3%) compared to the coronal region (25.5% ± 8.4%) (*p* > 0.05). In contrast, the immediately loaded group had lower percentages of Bone Area 1, with a more pronounced reduction in the apical region (15.2% ± 5.1%) compared to the coronal region (22.0% ± 13.1%) (*p* > 0.05). The amount of bone with medium mineralization (Bone Area 2) was also higher in the delayed‐loaded group, especially in the coronal region (36.5% ± 17.1%), while the immediately loaded group showed significantly lower trabecular bone percentages, with the coronal and apical values being 14.6% ± 2% (*p* < 0.05) and 11.1% ± 9.8% (*p* < 0.05), respectively. The immediately loaded group displayed a significantly greater proportion of highly mineralized cortical bone (Bone Area 3) in both the coronal (64.9% ± 16.5%) and apical (74.1% ± 15.6%) regions compared to the delayed‐loaded group (*p* < 0.05).

**TABLE 2:** Bone area percentages (mean ± SD) at selected interface region (within 100 µm).

	Delayed loaded (*n* = 16)		Immediately loaded (*n* = 5)	
	Coronal	Apical	Coronal	Apical
Bone Area 1—low mineralization (bone marrow)	25.5 ± 8.4aA	33.0 ± 13.3aA	22.0 ± 13.1aA	15.2 ± 5.1aA
Bone Area 2—medium mineralization (trabecular bone)	36.5 ± 17.1bA	12.5 ± 7.2bB	14.6 ± 2.0aB	11.1 ± 9.8aB
Bone Area 3—high mineralization (hard/cortical bone)	38.5 ± 19.6bA	53.5 ± 14.3cA	64.9 ± 16.5bB	74.1 ± 15.6bB

*Note*: Analyses performed on environmental scanning electron microscopy (ESEM) images at 1000× magnification considering the first 100 µm of bone located at implant body. Significant differences (*p* < 0.05) in bone area distribution are represented by different small letters (vertical column), and significant differences (*p* < 0.05) between regions of interest of loading groups are represented by different capital letters (horizontal row).

Table 3 reports the bone area percentages for the entire ROI (1.5 × 2.5 mm). Bone Area 2 was significantly higher in the delayed‐loaded group, especially in the coronal region (31.0% ± 13.7%), when compared to the immediately loaded group (11.6% ± 2.8%) (*p* < 0.05). Bone Area 3 was also significantly higher in the immediately loaded group (51.8% ± 15.5 in the coronal and 50.5% ± 10.6% in the apical region) compared to the delayed‐loaded group (32.2% ± 15.6% and 36.5% ± 12.1%) (*p* < 0.05).

**TABLE 3:** Bone area distribution percentages (mean ± SD) at selected ROI (1.5 × 2.5 mm).

	Delayed loaded (*n* = 16)		Immediately loaded (*n* = 5)	
	Coronal	Apical	Coronal	Apical
Bone Area 1—low mineralization (bone marrow)	35.8 ± 11.6aA	46.5 ± 9.7aA	44.1 ± 12.1aC	41.5 ± 10.1aC
Bone Area 2—medium mineralization (trabecular bone)	31.0 ± 13.7aA	17.1 ± 10.2bB	11.6 ± 2.8bB	8.9 ± 9.1aB
Bone Area 3—high mineralization (hard/cortical bone)	32.2 ± 15.6aA	36.5 ± 12.1aA	51.8 ± 15.5aB	50.5 ± 10.6aB

*Note*: Analyses performed on environmental scanning electron microscopy (ESEM) images at 100× magnification (ROI size 1.5 × 2.5 mm). Significant differences (*p* < 0.05) in bone area distribution are represented by different small letters (vertical column), and significant differences (*p* < 0.05) between ROI of loading groups are represented by different capital letters (horizontal row).

Abbreviation: ROI, region of interest.

**FIGURE 3:**
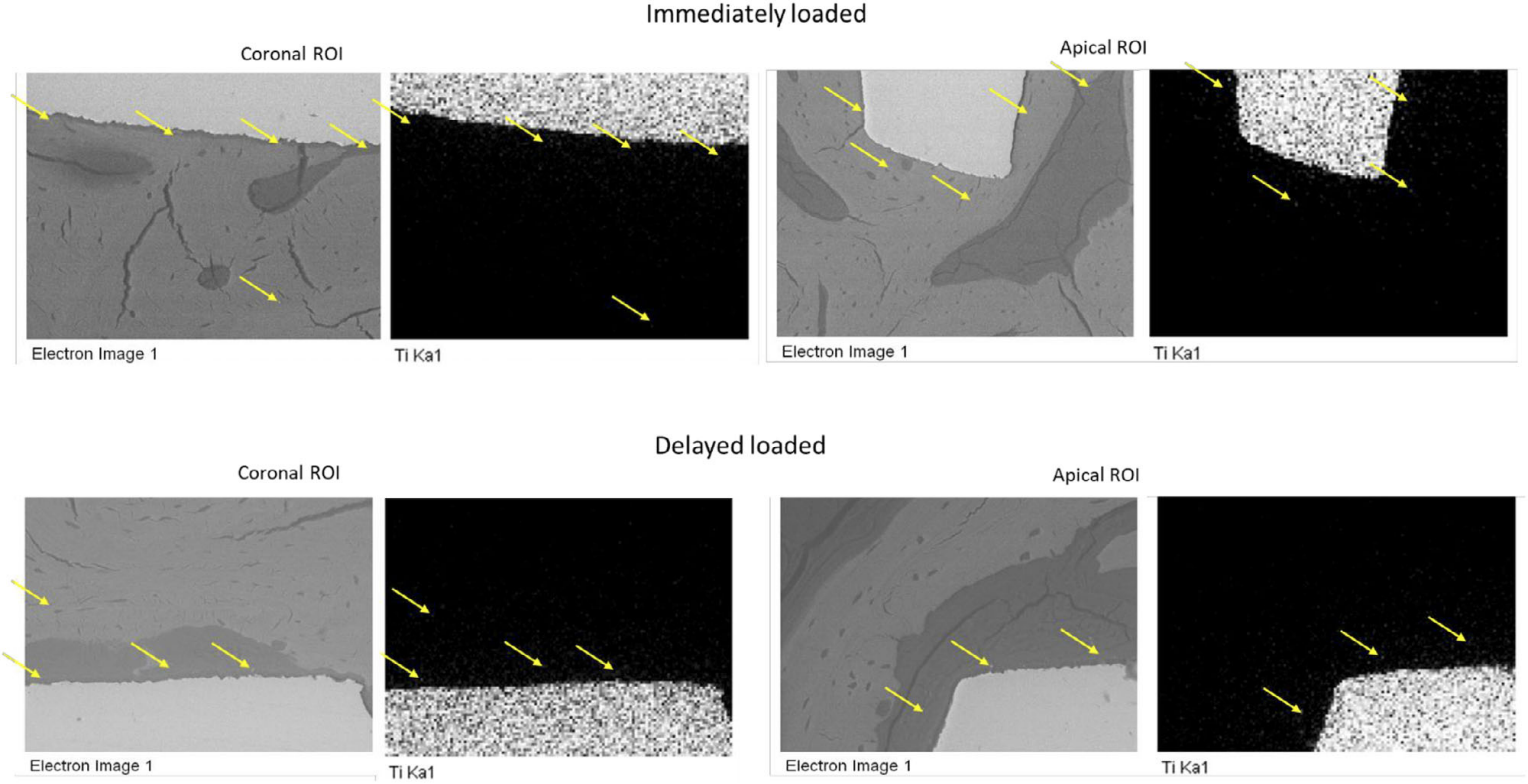
EDX elemental mapping (1000× magnification) on two representative implants taken at coronal and apical ROI from immediately loaded and delayed group. Yellow arrows highlight electron‐dense granules (light gray granules observed using ESEM and atomic Ti detected using EDX). Decreasing trend was observed in both implant groups, with lower presence of Ti at distant areas. EDX, energy‐dispersive X‐ray spectroscopy; ESEM, environmental scanning electron microscopy; ROI, region of interest; Ti, titanium.

Table 4 reports the Ti atomic percentages at different sites of delayed‐loaded and immediately loaded implants. Contact bone showed markedly higher atomic Ti percentages in immediately loaded biopsies (7.2 ± 5.1) when compared to the delayed‐loaded group (3.9 ± 2.4) (*p* < 0.05). No other significant differences between groups were observed. EDX analysis revealed a decreasing trend in Ti atomic content from the areas closest to the implant to the more distant regions (up to 2.0 mm). In control bone areas (apical portion, 3 mm from the implant body), no electron‐dense granules were detected on ESEM, and Ti atomic percentages were absent in EDX analysis, with significant differences in terms of atomic Ti percentages when compared to contact and remote bone areas (*p* < 0.05). EDX mapping confirmed this trend (Figure 3), supporting the absence of Ti migration in these regions.

**TABLE 4:** Atomic titanium percentages at different implant sites.

	Contact bone (within 300 µm)	Remote bone (300 µm–2.0 mm)	Distant bone (over 2.0 mm)	Apical bone (control)
Delayed loaded	3.9 ± 2.40aA	1.9 ± 3.4aB	0.29 ± 0.05aC	0.00aD
Immediately loaded	7.2 ± 5.1bA	2.3 ± 0.7aB	0.00bC	0.00aC

*Note*: Significant differences (*p* < 0.05) are represented by different small letters (vertical column), and significant differences (*p* < 0.05) between regions of interest of loading groups are represented by different capital letters (horizontal row).

**FIGURE 4:**
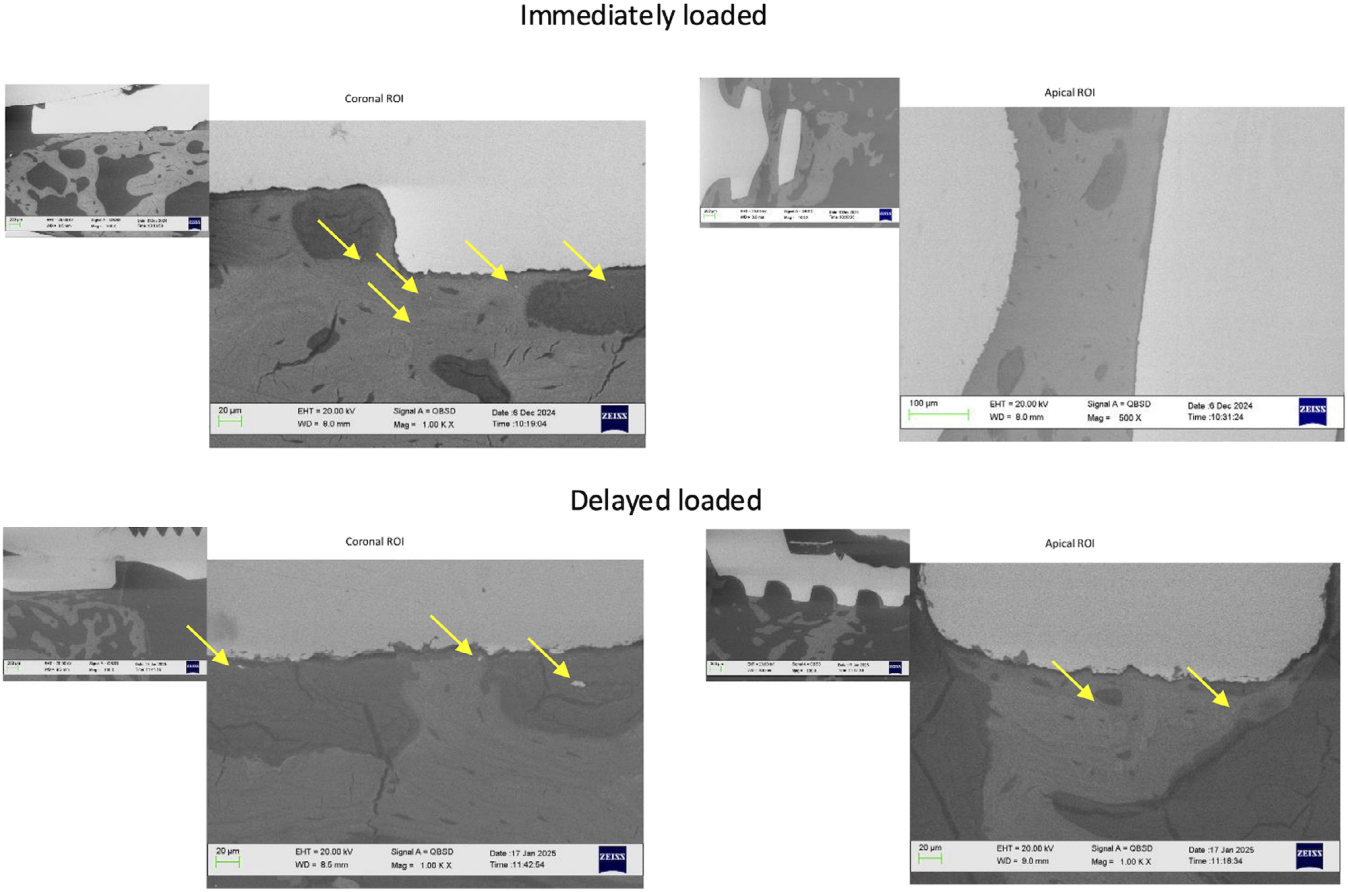
ESEM images (1000× magnifications) on coronal (first thread with bone tissue) and apical (last thread with bone tissue) ROI of one immediately loaded and one delayed‐loaded implant. Titanium granules (yellow arrows) were well detectable in coronal ROI of both groups, while apically, no or very few granules were observed. These granules were mostly detectable in low electron‐dense areas (Bone Areas 1 and 2), areas with low mineralization values. EHT, electron high tension; ESEM, environmental scanning electron microscopy; Mag, magnification; ROI, region of interest; WD, working distance.

Ti granules (2–10 µm) were identified in all analyzed regions, with a higher prevalence in the cortical and middle portions of the peri‐implant bone. These granules were more easily detectable in areas with low electron density. Bone tissue located distant to the implant exhibited a lower prevalence or no presence of Ti granules, as reported in Figure 4.

## 4 DISCUSSION

The analysis of bone histology by OM provides information on peri‐implant bone morphology but fails to deliver information on bone mineralization level, such as shifts in Ca/P ratios from carbonated apatite or changes in N content within the bone matrix (Ca/N and P/N ratios). Furthermore, the traditional histological approach does not detect the presence of external elements like metal ions or micrometric particles, which require additional and more invasive methodologies. Histological sectioning may also introduce height separation artefacts at the bone–implant interface, which makes it difficult to analyze this interface accurately.^34^

This study analyzed the bone mineralization degree and occurrence of Ti particle migration around implants placed in *M. fascicularis* in different loading conditions. This animal model has close similarities with human bone and could be used to assess soft tissue conditions^35^ and bone‐to‐implant contact^32^ or to detect metals in body regions far from implant rehabilitations.^36^ This study was performed using previously prepared ground sections from an earlier histomorphometric analysis.^31^ This approach allowed the application of ESEM‐EDX analyses, but future studies would benefit from prospectively designed experiments that allow for targeted analysis of bone remodeling and Ti particle migration under controlled conditions.

The en bloc removal of the mandible with implants was chosen over less invasive retrieval methods (e.g., trephine biopsy) because the intention was also to analyze Ti particle migration in distant regions, including apical bone sites that were not involved in implant placement or surgical manipulation. These distant sites were used as controls to confirm that Ti particle dissemination was not an artifact of the surgical procedure or sampling technique. In a previous study, the use of trephines and other cutting instruments introduced additional metallic contamination (such as iron or aluminum).^27^

The study revealed markedly higher atomic percentages of Ca and P but a similar Ca/P mineralization ratio when compared to previous human studies on retrieved bone block biopsies containing dental implants.^27^, ^28^ These data reflect more mineralized tissue in the animal model compared to human samples. A similar behavior in Ca and P atomic content was also noted in previous studies analyzing dog mandibular samples in different conditions^37^ or in sheep bone models.^38^

### 4.1 Mineralization degree around dental implants

During the remodeling process, osteoblasts synthesize new osteoid matrix and regulate bone mineralization by controlling Ca and phosphate (PO_4_) concentrations, leading to the formation of amorphous calcium phosphate,^4^, ^39^, ^40^ which serves as a precursor to biological carbonated hydroxyapatite.^41^, ^42^, ^43^ Osteoclasts, on the other hand, mediate bone resorption through the production of enzymes that degrade the organic matrix and mobilize bone apatite.^2^ As a consequence, bone remodeling results in significant variations in both mineral (Ca and phosphates) and organic (bone matrix, primarily collagen‐1) content.

Our ESEM‐EDX analysis allowed to identify distinct electron density areas, reflecting different bone mineralization behaviors between the loading groups (see Table S1 in online *Journal of Periodontology*). The variations of atomic Ca, P, and N percentages and proportions (i.e., Ca/P, Ca/N, and P/N ratios) were used as an index for hydroxyapatite maturation and bone mineralization. Nitrogen (N) was used instead of carbon (C) due to the abundant presence in bone as carbonated hydroxyapatite and also due to the presence in resins used to prepare the histological samples.^27^ Bone with low electron density (Bone Area 1, dark gray) with low Ca and P indicates poorly mineralized trabecular spaces. Bone with medium electron density (Bone Area 2, gray), with intermediate Ca and P and higher Ca/N and P/N, represents remodeling or immature bone. Bone with high electron density (Bone Area 3, light gray), richer in Ca and P, was identified as mature cortical bone. Bone Area 4 (light gray) was only observed in areas distant from implant surgeries and represented the control bone tissue. This area, richer in Ca, P, and N, evidenced low standard deviation values for all atomic ratios (see Table S1), indicating areas with stable bone tissue with no remodeling events.

The previous histomorphometric study on *M. fascicularis* biopsies revealed similar bone implant contact between the two loading groups and no significant differences.^31^ However, our study revealed some differences in bone area distributions when we took loading groups, bone ROI, and interface bone into account. When considering the entire bone tissue (Table 3), the differences are mostly evident in the coronal ROI. In these regions, bone remodeling areas (Bone Area 2) are more frequent in delayed‐loaded implants (*p* < 0.05) when compared to immediately loaded implants, while the apical ROI of both groups revealed a high presence of compact and mineralized bone (Bone Area 3), some trabecular bone areas (Bone Area 1), and limited bone remodeling areas (Bone Area 2).

However, when analyses were focused on the interface bone (bone tissue within 100 µm) (Table 2), a higher presence of Bone Area 3 and lower presence of Bone Area 1 was observed in the immediately loaded implant groups. This means that a more mature and compact bone is tightly attached to the apical region of the immediately loaded implants, while a higher presence of less mineralized bone is in contact with the apical region of delayed‐loaded implants. The different loading protocols could explain this behavior (immediate load vs. delayed load). Higher torque values at the moment of insertion and longer loading time in immediately loaded implants could have induced the formation of a different histological architecture with a different mineralization pattern.

Previous studies analyzed the effect of implant loading procedures on bone remodeling patterns around loaded versus unloaded implants^44^ and on mandibular bone retrieved post mortem.^28^ A previous ESEM‐EDX investigation on retrieved mandibular bone biopsies containing implants that had been loaded after 4 months revealed a higher presence of remodeling bone (Bone Area 2) and a lower presence of highly mineralized bone with respect to unloaded implants.^44^ A previous ESEM‐EDX investigation on retrieved mandibular bone block biopsies containing 9‐month loaded implants showed low presence of remodeling bone in coronal regions (Bone Area 2) and higher presence of mature mineralized bone (Bone Area 3).^28^ These findings support the concept that loading induces changes in mineralization and significantly affects remodeling in the first months. Histomorphometric studies on long‐term retrieved implants—more than 10 years—demonstrated peri‐implant bone stability and limited presence of remodeling areas around sound and stable implants.^45^

A gap between implant and bone tissue has been observed in a number of histological studies. This gap has been reported in several previously published histological and histomorphometrical analyses.^8^, ^27^, ^28^, ^29^, ^30^

Two main hypotheses were put forward. It was proposed that the bone–implant interface zone is not exclusively composed of cortical bone. It also includes implant marrow‐type tissue and nonmineralized regions.^34^

Nonmineralized extracellular matrix is initially laid down directly at the implant surface, with subsequent mineralization in relation to the implant bone stress transfer.^6^, ^26^, ^34^

However, this gap could also represent an artifact resulting from sample processing—specifically, the shrinkage of the embedding resin during fixation and dehydration steps—rather than a true biological feature of the bone–implant interface.^6^, ^26^

Several clinical studies provide evidence of radiographic signs of peri‐implant bone remodeling, also known as marginal bone loss (MBL), at the crestal portion of implants within the first 3–6 months after initial loading. This process is considered physiological as bone architecture undergoes structural rearrangements during the loading period and secondary osseointegration phases.^2^, ^3^, ^4^ Moreover, implants were placed at bone level or slightly subcrestal in both groups. This means that in the delayed‐loaded group, a second surgical procedure was required at 3 months to expose the implant neck and to proceed to the prosthetic phases. Second‐stage surgeries proved to induce higher bone loss 3–6 months after loading, and this could be the reason for a higher presence of Bone Area 2 in the coronal portion.^46^ However, sufficient plaque control is not possible in monkeys due to the need for general anesthesia for oral hygiene procedures.

### 4.2 Ti particle identification in analyzed samples

Another aim of the study was to investigate the presence of Ti particles in the bone tissues. Ti was identified in the peri‐implant bone as both granules and characteristic peaks in EDX spectra. These granules were predominantly found within the first 50 µm of the bone–implant interface, especially in the more coronal regions and in bone areas with low mineralization (Bone Areas 1 and 2).

The coronal and middle portions are affected by high mechanical stress during surgical insertion.^17^ The detection of Ti particles in these regions highlights the mechanical influence of implant placement on particle migration and deposition. The Ti detected was not due to external contaminants (histological processing phases) as no saws were used during histological processing.

The apical portion of the implant displayed markedly different characteristics. This region was not subjected to surgical preparation or significant stress during implant placement. As a result, Ti concentration was low in the apical portion of the implant and absent in the most apical bone regions (3 mm from implant apical threads), both as granules and in EDX spectra and elemental mapping. These findings agree with finite element analysis (FEA) studies, which demonstrate that stress levels are considerably lower in the apical regions compared to the coronal and middle areas.^47^

Ti particles and ions are released from dental implants due to various factors, including electrochemical interactions, friction, microbial activity, the host's inflammatory response and its byproducts, and chemical influences from the surrounding macro‐ and microenvironments.^48^, ^49^ During implant placement, significant changes occur on the implant surface: Insertion into the mandible can crack the stable oxide film, releasing Ti particles of different sizes and properties,^19^ causing slight fractures and compression in the adjacent bone.

Friction and torsional forces during the procedure also lead to wear and cracks—especially at the implant's coronal portion and threads. Additionally, deformation and wear of Ti implant tools during site preparation may further detach Ti particles,^17^ and drill bit residues could be detected in bone or water fluids during osteotomy, as confirmed by previous scanning electron microscopy (SEM) and EDX analyses.^50^

The role of loading in Ti migration in peri‐implantitis etiopathogenesis remains an important consideration. Immediate loading may exacerbate the release and migration of Ti particles due to mechanical stress at the bone–implant interface. In contrast, delayed loading allows more time for initial healing and bone remodeling, potentially reducing the extent of Ti particle dissemination. It remains unclear whether loading is the primary factor influencing Ti migration or rather one of several contributing factors with potentially less impact. The implant surface characteristics—such as surface treatment and roughness—may play a more critical role in particle release and dissemination.^51^ In the present investigation, we found a different trend between the two loading groups using the same implant with the same surface topography.

The role of Ti particles in bone has been often debated in the literature as a potential inflammatory factor that could trigger bone remodeling events due to the frequent detection of Ti granules in peri‐implantitis‐affected retrieved implant histologies.^52^ Some studies evidenced a potential additional risk for bone remodeling, etiological factors of released Ti in bone, leading to a higher risk of sudden bone loss and peri‐implantitis.^15^ Other studies evidenced the ubiquitous presence of such particles in healthy bone tissues, with no additional risks for bone loss around osseointegrated implants.^17^, ^27^

The association between presence of nanoparticles and peri‐implant pathology remains unspecific. The tendency to find more Ti in close proximity to the implant surface in specimens from diseased sites could be the consequence of release caused by the activity of inflammatory cells and bacteria that are present in peri‐implant lesions.^20^

The present study evidenced a ubiquitous presence of Ti particles (electron‐dense granules detected using ESEM) and ions (detected using EDX analysis and mapping) in bone areas with different mineralization and histological morphology. In fact, it was easier to identify Ti particles in areas with low mineralization due to the high electron density of Ti. However, elemental mapping and EDX spectra revealed some Ti also in highly mineralized tissues.

A limitation of the study could be the imbalance in loading group sizes, with only six implants subjected to immediate loading. This small sample size reduces the statistical power to detect significant differences between immediate‐loading and nonimmediate‐loading groups, particularly for bone mineralization parameters. As a result, the findings should be interpreted with caution.

## 5 CONCLUSIONS

This study demonstrated the presence of Ti particles in peri‐implant bone tissues of *M. fascicularis*, particularly in coronal regions subjected to higher mechanical stress. ESEM‐EDX analysis revealed distinct areas of bone mineralization, with greater apical mineralization observed in immediately loaded implants compared to delayed loading. Bone remodeling activity (Bone Area 2) was predominantly observed in the coronal regions of implants. The apical portion of immediately loaded groups presented a higher rate of mineralization when compared to the delayed‐loaded group. These findings suggest that both loading protocol and mechanical stress may influence Ti nanoparticle migration and mineralization around implants in this animal model.

## AUTHOR CONTRIBUTIONS

*Conceptualization*: Carlo Prati and Georgios Romanos. *Methodology*: Carlo Prati and Fausto Zamparini. *Validation*: Maria Giovanna Gandolfi. *Formal analysis*: Fausto Zamparini. *Investigation*: Chooi Gait Toh and Georgios Romanos. *Resources*: Georgios Romanos, Chooi Gait Toh, and Carlo Prati. *Data curation*: Fausto Zamparini and Andrea Spinelli. *Writing—original draft preparation*: Fausto Zamparini, Carlo Prati, and Georgios Romanos. *Writing—review and editing*: Maria Giovanna Gandolfi, Stefano Chersoni, Giovanni Badiali, and Achille Tarsitano. *Visualization*: Andrea Spinelli and Fausto Zamparini. *Supervision*: Georgios Romanos and Carlo Prati.

## CONFLICT OF INTEREST STATEMENT

The authors declare no conflicts of interest.

## FUNDING INFORMATION

The authors received no specific funding for this work.

## ETHICS STATEMENT

This study was approved by the animal ethics committee of the University of Malaya, Kuala Lumpur, Malaysia (Ethics Ref. No.: U1/10/6/96/TCG (R)‐1) and supported financially by a grant from the Ministry of Science, Technology, and Environment, Malaysia. The study was also supported by a grant from the Master's program in Clinical Endodontics, University of Bologna, Italy.

## Supporting information



Supporting Information

## Data Availability

The data that support the findings of this study are available upon reasonable request.
