# Supplementary material for: Titanium migration and bone response in loaded osseointegrated implants: ESEM‐EDX analysis in Macaca fascicularis
Source: J Periodontol. 2025 Sep 11;97(3):527–39. doi: 10.1002/jper.70003 (PMC13111777; doi:10.1002/jper.70003)
Supplement: Supplementary file 1 — Supporting Information [file JPER-97-527-s001.docx]

**Supplementary Material**

**Title: Titanium Migration and Bone Response in Loaded Osseointegrated Implants: ESEM-EDX Analysis in *Macaca Fascicularis***

**Authors:** Fausto Zamparini ^1,2^, Andrea Spinelli ^1,2^, Maria Giovanna Gandolfi ^2^, Stefano Chersoni ^1^, Achille Tarsitano ^3^, Giovanni Badiali ^3^, Chooi Gait Toh ^4^, Carlo Prati ^1^, Georgios Romanos* ^5,6^

**Affiliations:**

1. Endodontic Clinical Section, Dental School, DIBINEM, University of Bologna, Bologna, Italy
2. Laboratory of Biomaterials and Oral Pathology, Dental School, DIBINEM, University of Bologna, Bologna, Italy
3. Oral and Maxillo-Facial Surgery Unit, DIBINEM, University of Bologna, Bologna, Italy
4. School of Health Sciences, International Medical University, Kuala Lumpur, Malaysia
5. Department of Periodontics and Endodontics, School of Dental Medicine, Stony Brook University, Stony Brook, NY, United States
6. Department of Oral Surgery and Implant Dentistry, Dental School (Carolinum), Johann Wolfgang Goethe University, Frankfurt, Germany

Immediately loaded group


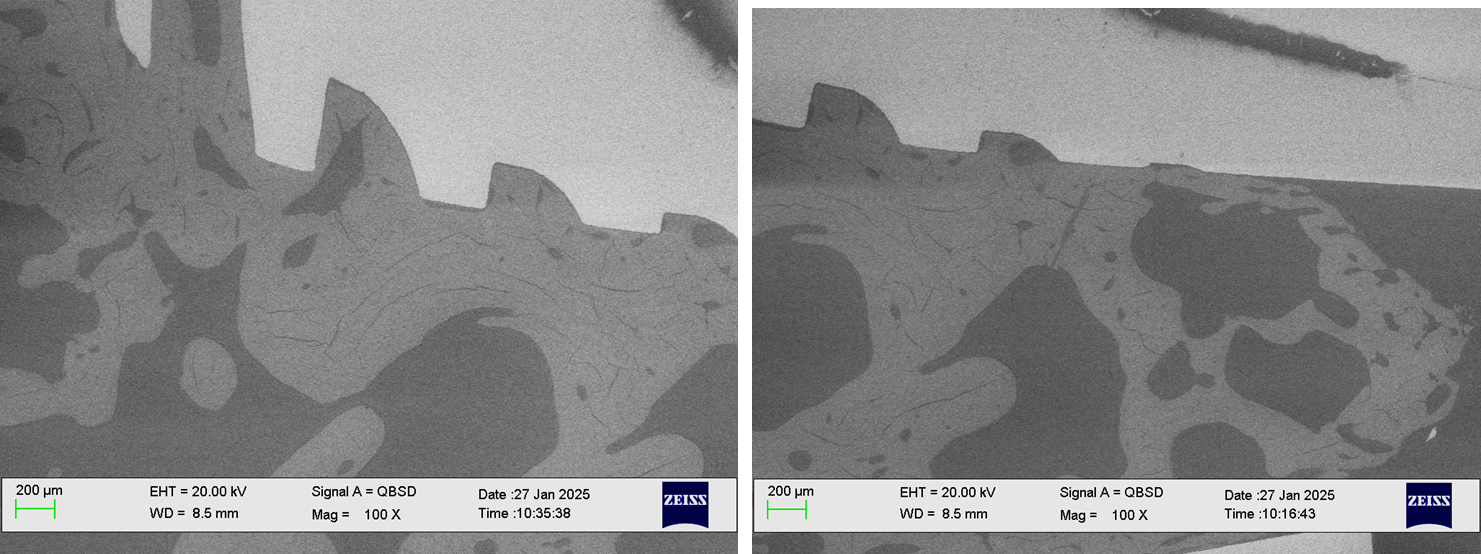


Delayed loaded group


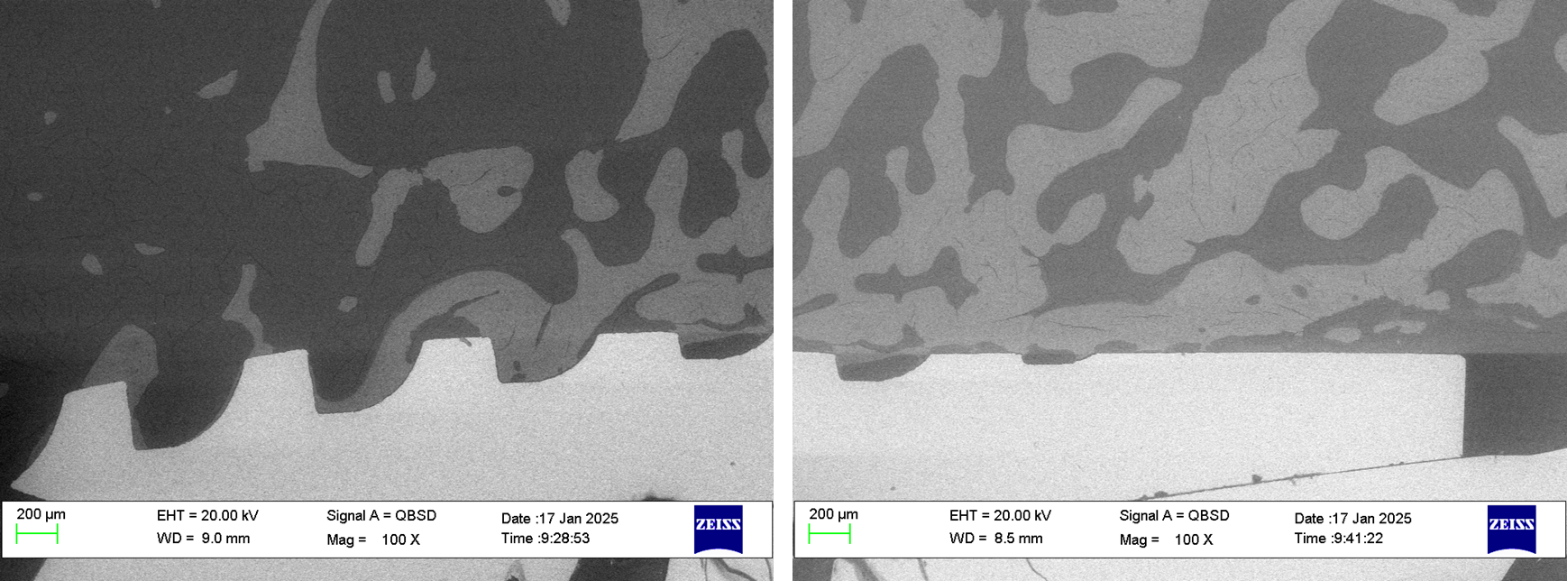


**Figure S1.** ESEM images (100x magnification) of two representatives immediately loaded and delayed loaded implant biopsies. Contact Bone tissue (within 100-300 µm from the threads) around immediately loaded group revealed compact and electron dense bone with few areas of remodeling (and less electron dense bone) when compared to the delayed loaded implant. Delayed group showed a higher percentage of bone marrow areas (low electron dense areas), at both coronal and apical ROIs.

| Table S1 | Analysis of bone areas (Mean±SD) according to EDX elemental content | | | |
| --- | --- | --- | --- | --- |
|  | | Ca | P | N |
| Bone Area 1 low mineralized | | 0.99 ±0.33 | 0.81 ±0.26 | 4.08±1.3 |
| Bone Area 2 medium mineralized | | 3.49 ±2.07 | 2.65 ±1.56 | 5.35±3.22 |
| Bone Area 3 highly mineralized | | 5.27 ±1.54 | 3.99 ±1.11 | 7.35±1.80 |
| Bone Area 4 control bone distant from surgical area | | 6.91 ±1.12 | 4.98 ±0.75 | 12.07±1.20 |
|  | | Ca/N | P/N | Ca/P |
| Bone Area 1 low mineralized | | 0.30±0.14 | 0.24±0.09 | 1.22±0.08 |
| Bone Area 2 medium mineralized | | 0.67±0.18 | 0.50±0.13 | 1.31±0.07 |
| Bone Area 3 highly mineralized | | 0.77±0.43 | 0.58±0.31 | 1.30±0.05 |
| Bone Area 4 control bone distant from surgical area | | 0.51±0.02 | 0.37±0.08 | 1.38±0.06 |
